# Network Pharmacology and Molecular Docking Verify the Mechanism of Qinshi Simiao San in Treating Chronic Prostatitis in the Rat Model

**DOI:** 10.1155/2022/7098121

**Published:** 2022-01-12

**Authors:** Chenxi Li, Lei Xu, Xuyao Lin, Qingrui Li, Shaoming Liu, Lipeng Fan, Wei Fu, Feiyu Liu, Zhuojun Yuan, Guozheng Qin

**Affiliations:** ^1^Yunnan University of Chinese Medicine, Kunming 650500, China; ^2^Aerospace Central Hospital, Beijing 100049, China; ^3^Nanjing University of Chinese Medicine, Nanjing 210023, China; ^4^Shenzhen Baoan Hospital of Traditional Chinese Medicine (Group), Shenzhen 518133, China; ^5^The First Affiliated Hospital of Yunnan University of Chinese Medicine, Kunming 650021, China

## Abstract

**Background:**

Using network pharmacology and molecular docking, this study aimed to explore the active pharmaceutical ingredients (APIs) and molecular mechanism of Qinshi Simiao San (QSSMS) in the treatment of chronic prostatitis (CP) and verify our findings in the rat model.

**Methods:**

The APIs of QSSMS and the common targets of QSSMS and CP were screened from the TCMSP database. The STRING database and Cytoscape software were used to construct the network graph. The enriched GO and KEGG pathways were displayed by David software and R software. Molecular docking was performed to visualize key components and target genes. In addition, the rats model of CP was established to verify the molecular mechanism of QSSMS.

**Results:**

Network pharmacology showed that the APIs of QSSMS mainly included quercetin, kaempferol, formononetin, isorhamnetin, and calycosin. QSSMS alleviated CP mainly through the negative regulation of the apoptotic process, oxidation-reduction process, inflammatory response, and immune response. Molecular docking showed that the APIs could bind to the corresponding targets. QSSMS repaired the pathological damage of prostate tissue, upregulated the expression of oxidative stress scavenging enzymes CAT and SOD, and downregulated the peroxidative product MDA, inflammatory factors IL-1*β*, IL-6, TNF-*α*, COX-2, PGE2, and NGF, and immune factors IgG and SIgA.

**Conclusion:**

The APIs in QSSMS may inhibit inflammation in the rat CP model by regulating immune and oxidative stress.

## 1. Introduction

Chronic prostatitis (CP) is a common disease in the urinary system, accounting for more than 90% of the total cases of prostatitis. CP mainly occurs in men aged 20 to 50 years [1, 2], with an incidence of 8.4% in China [3]. Its manifestations include pain in the perineum, lower abdomen, and other parts and abnormal urination. CP may lead to sexual dysfunction and anxiety [4, 5]. Premature ejaculation appears in 45.3% and erectile dysfunction in 47.4% of CP patients [6]. CP also decreases sperm quality and weakens males' reproductive function [7].

Inflammation, abnormal immune response, oxidative stress injury, and endocrine disorders are all closely related to the occurrence of CP [8]. Tissue damage caused by prostatitis may disrupt the imbalance between reactive oxygen species and antioxidants [9]. Antibiotics, nonsteroidal anti-inflammatory drugs, *α*-receptor blockers, and other drugs are clinically used to treat CP, but their efficacy is often unsatisfactory.

Qinshi Simiao San (QSSMS) is a prescription for the clinical treatment of CP invented by Professor Qin Guozheng, a famous TCM doctor in China. In this prescription, four ingredients (*Huang Qi, Jin Yin Hua, Xuan Can,* and *Gan Cao*) are used to exert an effect of “eliminating, supporting, and tonifying” [10]. As a bidirectional immune regulator, *Huang Qi membranaceus* contains polysaccharide, saponin, flavone, and so on [11]. *Huang Qi* extract can significantly reduce the expression of TNF-*α*, IL-1*β*, COX-2, PEG2, and TGF-*β*_1_ in the prostate tissue of rats with nonbacterial CP [12]. *Xuan Can* extract exerts obvious anti-inflammatory and analgesic effects on mice [13]. Phenolic acid is the main component of *Jin Yin Hua* with a strong anti-inflammatory effect [14]. Liquiritigenin is a kind of flavonoid compound extracted from *Gan Cao*, with evident antioxidative and anti-inflammatory effects [15]. Liquiretin effectively inhibits the inflammatory response through upregulating estrogen receptor-*β* (ER-*β*) [16].

In recent years, network pharmacology has been widely used in mechanistic research on drugs. In this study, a pharmacological network was used to predict the active pharmaceutical ingredients (APIs), potential targets, and signaling pathways of QSSMS in the treatment of CP. The molecular docking was used to verify the associations between APIs and targets. The efficacy and mechanisms of QSSMS were verified in a rat model of CP established through the injection of carrageenan. Our findings will provide a theoretical basis for the TCM treatment of CP.

## 2. Material and Methods

### 2.1. Screening the APIs and Targets of QSSMS

“Huang Qi,” “Jin Yin Hua,” “Xuan Can,” and “Gan Cao” were retrieved from the Traditional Chinese Medicine Systems Pharmacology (TCMSP) database (https://tcmspw.com/tcmsp.php). Admin parameters were set as oral bioavailability (OB) >30% and drug-likeness (DL) >0.18 to obtain the APIs of QSSMS. In the UNIPROT database, the targets of the APIs of QSSMS were annotated.

### 2.2. Collecting CP Targets

GeneCards, DisgeNet, and Online Mendelian Inheritance in Man (OMIM) databases were used to screen the targets of CP. The search was conducted using the term “Chronic Prostatitis.” The results retrieved from the three databases were summarized.

### 2.3. Screening Common Targets and Constructing a Protein-Protein Interaction (PPI) Network

Through Venny2.1 software, the targets of the APIs of QSSMS and those of CP were mapped to screen out the overlapped ones. The Venn plot was drawn. The Search Tool for the Retrieval of Interacting Genes/Proteins (STRING) database was used to retrieve the common targets through the entry of “Multiple proteins.” The default target score was set as > 0.41, and no free targets were allowed to present. The PPI network and TSV files were generated, and the topology analysis of the network was performed using Cytoscape3.8.2 software. Key targets in the network were visualized.

### 2.4. Gene Ontology (GO) Enrichment and Kyoto Encyclopedia of Genes and Genomes (KEGG) Pathway Enrichment Analyses

DAVID software (https://david.ncifcrf.gov) was used to search for enriched terms in three aspects: biological process (BP), cellular component (CC), and molecular function (MF). The results were visualized using Excel. The R software was used to identify the enriched KEGG pathways of common targets. The bubble plots were drawn for the top 20 pathways according to the corrected *P* values.

### 2.5. Preparation of QSSMS

Each dose (80 g) of QSSMS is composed of Huang Qi (30 g), Jin Yin Hua (10 g), Xuan Can (30 g), and Gan Cao (10 g) (all purchased from Yunnan Hospital of Traditional Chinese Medicine). According to the Pharmacological Experimental Methodology of Traditional Chinese Medicine, one dose of QSSMS was soaked in water (5 to 8 times in weight) at room temperature for 30 min, boiled for 30 min, and filtered. The residue was diluted 3 to 6 times with water, boiled for another 15 to 20 min, and filtered. Two volumes of decoction were combined and decocted again to extract APIs. The extract was stored at 4°C until use. The daily dose for humans was converted to a dose for rats based on the body surface and a conversion coefficient of 0.018. Considering 80 g as the medium dose for humans, the intragastric doses in QSSMS-L (Qinshi Simiao San low dose) group, QSSMS-M (Qinshi Simiao San medium dose) group, and QSSMS-H (Qinshi Simiao San high dose) group were calculated as 4 g/kg/d, 8 g/kg/d, and 16 g/kg/d, respectively.

### 2.6. Main Reagents and Instruments

Main reagents and instruments included Rat IL-1*β* ELISA Kit (ML037361, China); Il-6 RAT ELISA Kit (ML102828, China); Rat TNF-*α* ELISA Kit (Ml002859, China); Rat COX-2 ELISA Kit (Ml058808, China); Rat PGE2 ELISA Kit (ML003036, China); MDA Assay Kit (A003-1-2, Nanjing Jiancheng, China); SOD Assay Kit (A001-3-2, Nanjing Jiancheng, China); CAT Assay Kit (A007-1-1, Nanjing Jiancheng, China); Rat IgG ELISA Kit (ML003019, China); Rat SIgA ELISA Kit (Ml003136, China); NGF antibody (DF6061, affinity, USA); carrageenan (C1013-25G, Merck, USA); electric constant temperature blast drying oven (DGG-9140B, Shanghai Shenxin, China); high-speed refrigerated centrifuge (Thermo Scientific, USA); microplate reader (SPECTCA MAX190, Molecular, USA); fast mixer (MX-S, SCILOGEX, USA); upright fluorescence microscope (NIKON ECLIPSE C1, Nikon, Japan).

### 2.7. Animal Model and Treatment

Thirty healthy male Wistar rats with a bodyweight of 250 ± 20 g were divided into 5 groups by random number method (*n* = 6): Control group, Model group, QSSMS-L group, QSSMS-M group, and QSSMS-H group. The CP rat model was established using the method of Wang et al. [17, 18]. After adaptive feeding for 7 d, the rats were anesthetized. Then, 1% carrageenan (100 *μ*l) was injected with a 1 ml syringe into the ventral lobe of the prostate in the Model group, and the same amount of normal saline was injected in the Control group. After 7 d, the model was replicated, and the rats were given intragastric QSSMS powder at high, medium, and low doses, respectively. The Model group and the Control group were gavaged with the same amount of normal saline. After 28 days of intragastric administration, the rats were anesthetized with ether. The blood was sampled from the aorta and centrifugated for serum collection. The prostate tissue was sampled quickly. All animal experiments were approved by Yunnan University of Chinese Medicine Animal Ethics Committee (permission number: R-06202075) and were implemented complying with the Guidelines for the Care and Use of Laboratory Animals.

### 2.8. Hematoxylin and Eosin (HE) Staining

The tissue sample of prostate lateral lobes was paraffin-embedded and then cut into tissue blocks 5 ul thick for HE staining. The paraffin sections were routinely dewaxed, hydrated, and then washed with distilled water for 5 min and stained with hematoxylin for 8 min. To remove floating color, the tissue was washed with tap water for 2 s and then differentiated with 1% hydrochloric acid alcohol. When the tap water turned blue, the tissue was eosin-stained for 3 s at room temperature, and the tap water ceased to be colored. Dehydration was performed using alcohol with a gradient concentration of 75%, 80%, 85%, 90%, 100%, and 100% for 10 min. Xylene was transparent for 30 min. Neutral gum sealing and holographic scanning were conducted. The pathological changes such as infiltration of lymphocytes and neutrophils, regular shape of acinar, edema, dilation of the glandular lumen, widening of the interstitium, proliferation of fibrocytes, and angiogenesis were observed under a light microscope.

### 2.9. Immunohistochemistry

The expression of cyclooxygenase-2 (COX-2) and nerve growth factor (NGF) in the prostate tissue was determined by immunohistochemistry. A small piece of fresh prostate tissue was embedded, dewaxed, and dehydrated with gradient alcohol. After antigen retrieval, the tissue was washed with PBS three times, fixed in 4% paraformaldehyde for 20 min, washed again three times with PBS, then permeabilized with 0.2%TritonX100 for 10 min, and washed three times with PBS. After staining, the tissue was rinsed with 0.0l mol/L PBS (pH 7.4) three times and shaken occasionally. The tissue slices were sealed with buffered glycerin. The expression of COX-2 and NGF in rat prostate tissue was observed under a fluorescence microscope.

### 2.10. Reactive Oxygen Species (ROS) Staining

An appropriate amount of ipsilateral prostate tissue was chosen for ROS immunofluorescence staining. First, a histochemical pen was used to draw a circle around the tissue, followed by adding an autofluorescence quenching agent for 5 min and then washing with water for 10 min. ROS dye was added and incubated in the dark at 37°C for 30 min, washed with PBS three times, then added with DAPI solution, and incubated at room temperature in the dark for 10 min. After the slices were washed with PBS three times, the slices were slightly shaken dry, sealed with antifluorescence quenching sealing tablets, and then photographed under a microscope.

### 2.11. ELISA

The expression levels of IL-6, IL-1*β*, TNF-*α*, COX-2, prostaglandin E2 (PGE2), IgG, SIgA, malondialdehyde (MDA), superoxide dismutase (SOD), and catalase (CAT) were detected by ELISA. After anesthesia, 5 ml of blood was extracted from the abdominal aorta and placed in an anticoagulant tube. The blood was centrifuged at 4°C for 20 min (3000 RPM). The upper serum was taken and frozen at −80°C. At the same time, an appropriate amount of prostate tissue was cut and stored in PBS. The sample was fully homogenized with a homogenizer, and the rest of the samples were stored in a refrigerator at −80°C. The prostate tissue was centrifuged for about 20 min at 3000 RPM, and the homogenate supernatant was collected. The concentrations of IL-6, IL-1*β*, TNF-*α*, COX-2, PGE2, IgG, and SIgA in the prostate tissue and serum were determined by ELISA. Similarly, biochemical kits were used to detect the contents of MDA, SOD, and CAT in the supernatant of the prostate tissue. Methods and procedures were completed following the kit instructions.

### 2.12. Statistical Analysis

Results were expressed as mean and standard deviation. Intergroup differences were presented by one-way analysis of variance and graph-based test. All data were processed on GraphPad Prism 8.0 software, and a *P* < 0.05 was considered statistically significant.

## 3. Results

### 3.1. Target Prediction

**Figure 1:**
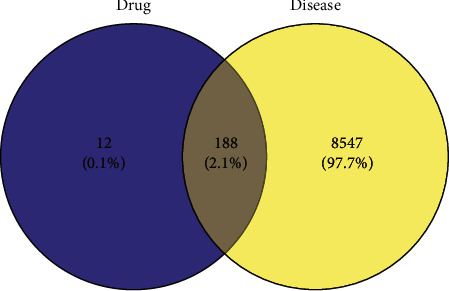
Common targets of QSSMS APIs and CP.

A total of 144 APIs in QSSMS were obtained from the TCMSP database, involving 1762 genes. Using the UNIPROT database, 200 target genes of those APIs were obtained. From GeneCards, DisgeNet, and OMIM databases, 8735 CP-related targets were obtained. Through Venny 2.1 software, the APIs and CP targets of QSSMS were mapped, and 188 common targets were screened out, as shown by the Venn diagram (Figure 1).

### 3.2. Analysis of PPI Network

**Figure 2:**
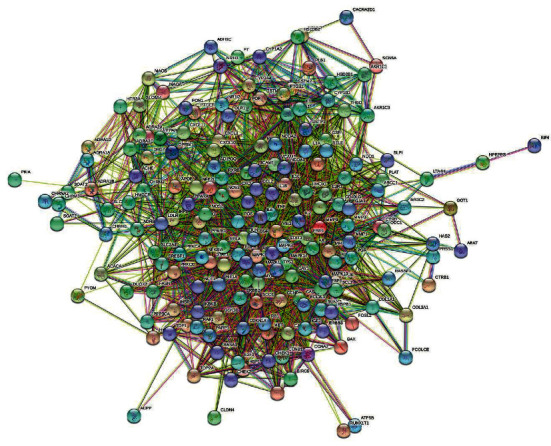
Analysis of PPI network.

**Figure 3:**
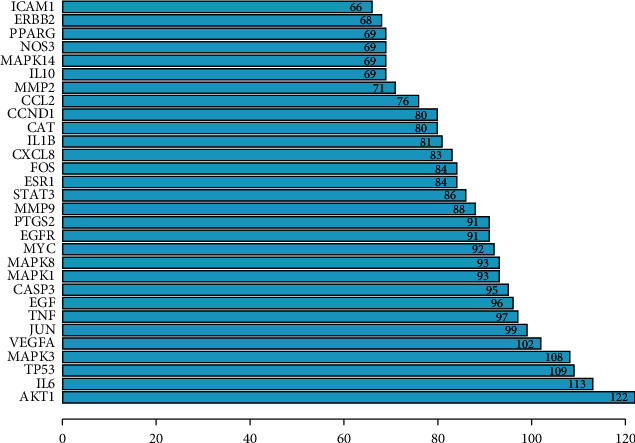
Filter key proteins in the network through barplot.

The overlapped targets of QSSMS and CP were imported into the STRING database to construct their PPI network (Figure 2). The network had 188 nodes and 3997 edges. According to the numbers of nodes connected with them, the top 10 proteins were AKT1, IL6, TP53, MAPK3, VEGFA, JUN, TNF, EGF, CASP3, and MAPK1 (Figure 3).

### 3.3. Drug-APIs-Genes-Disease Network

**Figure 4:**
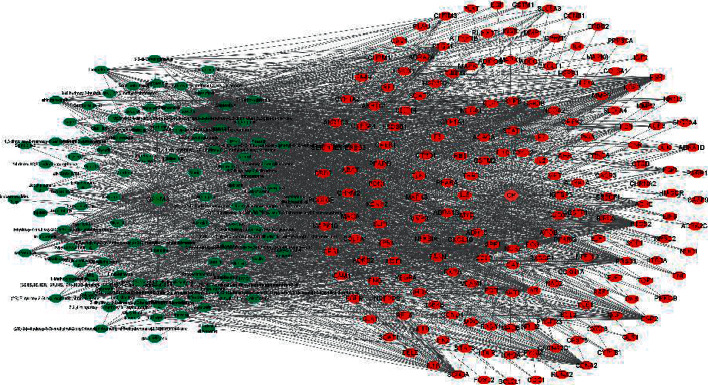
Network of QSSMS-APIs-Genes-CP.

The Cytoscape 3.8.2 software was used to build a Drug-APIs-Genes-Disease network (Figure 4). In this network, 188 genes were correlated with 144 APIs of QSSMS, with a total of 2,000 edges. The top 5 enriched APIs were quercetin, kaempferol, formononetin, isorhamnetin, and calycosin, suggesting that QSSMS APIs target diverse genes in the treatment of CP.

### 3.4. GO Functional Enrichment Analysis

**Figure 5:**
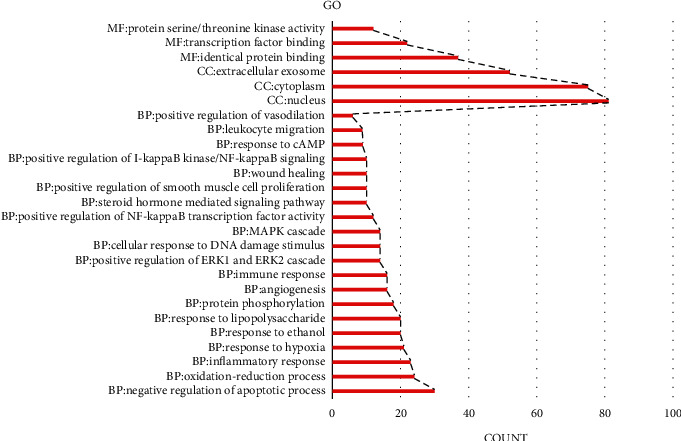
GO function enrichment analysis.

The 188 genes were subjected to GO analysis using David software. The results involved 678 BPs, 66 CCs, and 138 MFs (Figure 5). The BPs mainly involved negative regulation of the apoptotic process, oxidation-reduction process, inflammatory response, immune response. CCs mainly involved regulation of nucleus, cytoplasm, and extracellular exosome, and so on. MFs mainly involved the regulation of identical protein binding, transcription factor binding, protein serine/threonine kinase activity, and so on.

### 3.5. KEGG Pathway Enrichment Analysis

**Figure 6:**
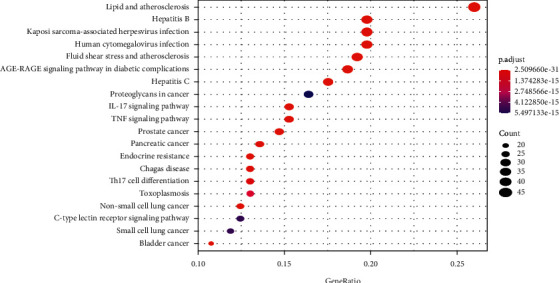
KEGG enrichment analysis.

KEGG enrichment analysis was carried out on the R software. A total of 175 enriched pathways were screened out. The top 20 most enriched pathways included the IL-17 signaling pathway, prostate cancer, bladder cancer, TNF signaling pathway, and endocrine resistance. These signaling pathways were involved in the regulation of inflammatory response, cell apoptosis, endocrine and prostate cancer, bladder cancer, and so on (Figure 6).

### 3.6. Molecular Docking Results

**Figure 7:**
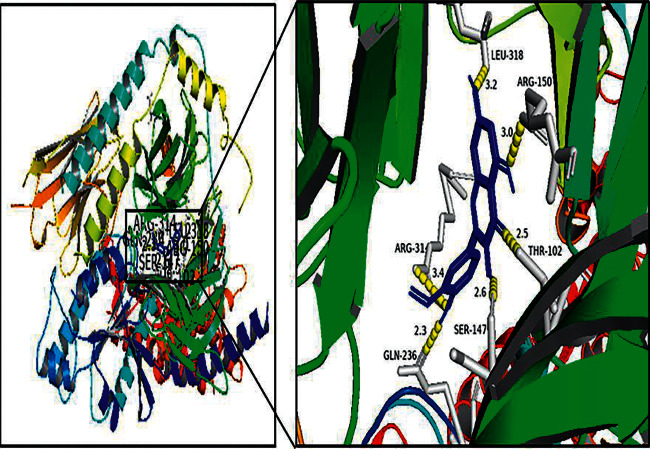
Molecular docking results of isorhamnetin and PGE2.

The software AutoDock Vina was used to carry out molecular docking between the top five APIs and the top five target genes. The default number of docking times was set as 9. It is generally believed that binding energy <−5.0 kcal/mol indicates good binding between one target protein and one API. The smaller the binding energy, the better the docking between the two. 84% of the docking scores were less than −5.0 kcal/mol, indicating that the tight link between them. Among them, isorhamnetin had the strongest association with PGE2 (Table 1 and Figure 7).

### 3.7. QSSMS Relieved the Pathological Injury

**Figure 8:**
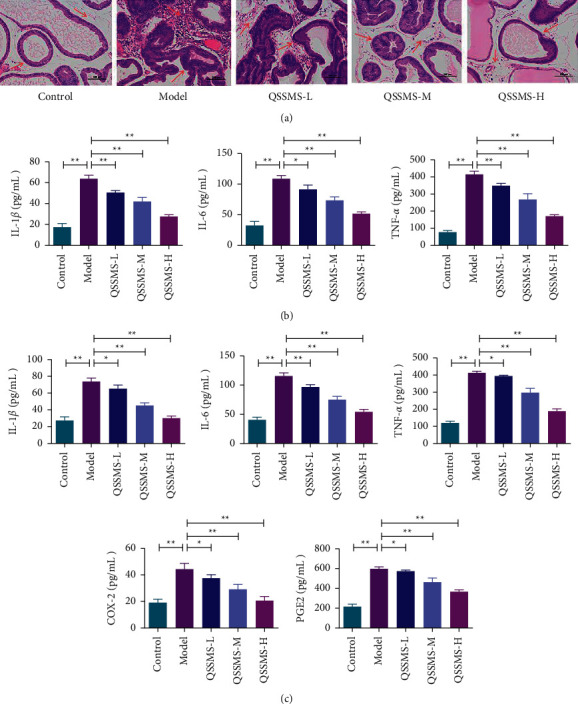
HE staining and ELISA (scale bar = 100 *μ*m). (a) HE staining of rat prostate tissue. (b) Expression of IL-1*β*, IL-6, and TNF-*α* in rat serum. The data are shown as the mean ± SEM of three independent experiments. (c) Expression of IL-1*β*, IL-6, TNF-*α*, COX-2, and PGE2 in the prostate tissue. The data are shown as the mean ± SEM of three independent experiments (no significant difference in NS, ^*∗*^*P* < 0.05, and ^*∗∗*^*P* < 0.01).

In our CP model, severe inflammatory infiltration was observed in the prostate tissue of rats at 7 d after carrageenan induction [19]. The morphology of prostate tissue was evaluated after HE staining. We found significant lymphocyte infiltration, decreased acinar diameter, dilated glandular lumen, and interstitial edema in the Model group, which confirmed the successful replication of the model. After 28 d of treatment with QSSMS, the glandular lumen structure of prostate tissue was restored, the infiltration of inflammatory cells in the glandular lumen and interstitial space was inhibited, the interstitial space was narrowed, and the proliferation of fibrocytes and blood vessels was reduced in a dose-dependent manner. The improvement was the most obvious in the QSSMS-H group. These findings confirmed the effect of QSSMS in relieving CP from the perspective of tissue morphology (Figure 8(a)).

### 3.8. QSSMS Inhibited the Expression of Inflammation-Related Factors

**Figure 9:**
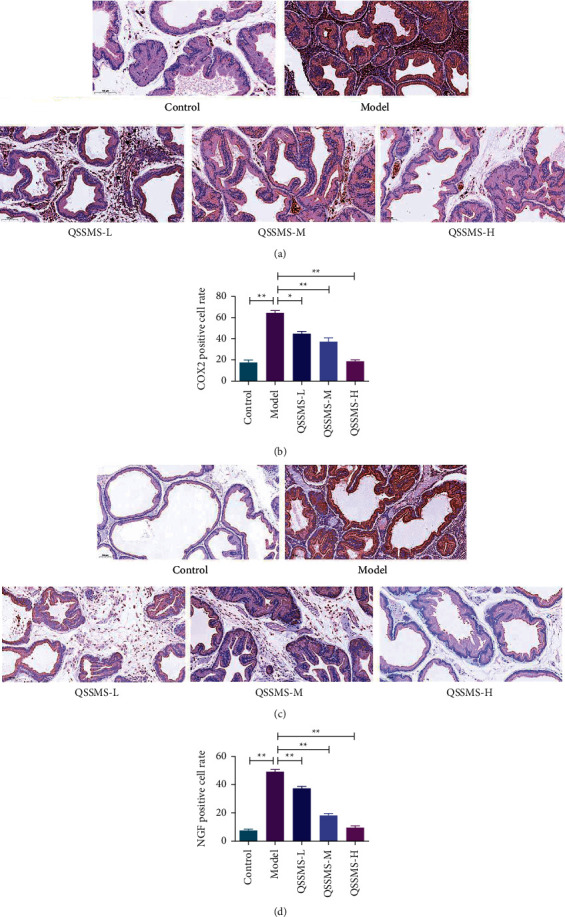
COX-2 and NGF were detected by immunohistochemistry. (a) Immunohistochemical results of COX-2 and NGF in the prostate tissue (scale bar = 100 *μ*m). (b) Positive rate of COX-2 in the prostate tissue. (c) Immunohistochemical results of NGF in the prostate tissue (scale bar = 100 *μ*m). (d) Positive rate of NGF in the prostate tissue. The data are shown as the mean ± SEM of three independent experiments. The data are shown as the mean ± SEM of three independent experiments (no significant difference in NS, ^*∗*^*P* < 0.05, and ^*∗∗*^*P* < 0.01).

After the CP model was successfully replicated in rats, the expression of inflammatory factors in the prostate tissue and serum of rats was detected by ELISA. It was observed that the expression levels of IL-6, IL-1*β*, TNF-*α*, and PGE2 in the Model group were significantly higher than those in the Control group (*P* < 0.05) (Figures 8(b) and 8(c)). Immunohistochemistry detected that the expression levels of COX-2 and NGF in the Model group were also increased (*P* < 0.05) (Figure 9). After 28 d of QSSMS treatment, the expression levels of IL-6, IL-1*β*, TNF-*α*, and PGE2 in prostate tissue and serum were significantly decreased, compared with those in the Control group (*P* < 0.05) (Figures 8(b) and 8(c)). The expression levels of COX-2 and NGF showed a downward trend (*P* < 0.05) (Figure 9).

### 3.9. QSSMS Alleviated Oxidative Stress-Induced Injury

**Figure 10:**
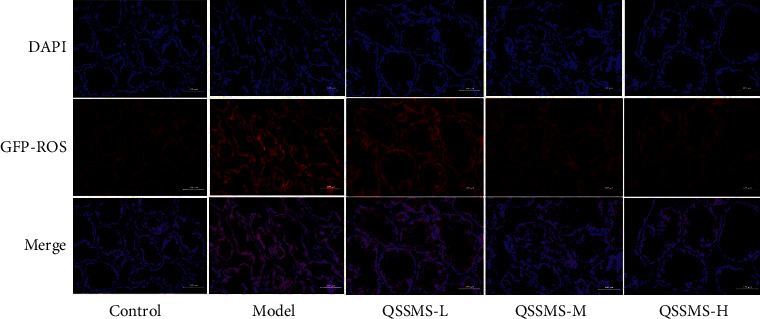
ROS staining in prostate tissue (scale bar = 100 *μ*m).

**Figure 11:**
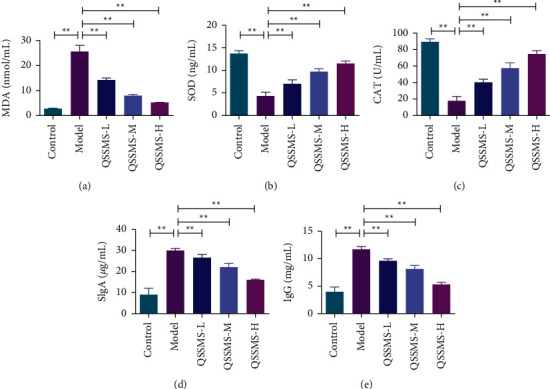
The expression of oxidative stress markers in prostate tissue and immune factors in serum were detected by ELISA. (a) Expression results of MDA in the prostate tissue. (b) Expression results of SOD in the prostate tissue. (c) Expression results of CAT in the prostate tissue. (d) Expression results of SIgA in the prostate tissue. (e) Expression of IgG in the serum. The data are shown as the mean ± SEM of three independent experiments (no significant difference in NS, ^*∗*^*P* < 0.05, and ^*∗∗*^*P* < 0.01).

Oxidative stress and immunity are closely related to the occurrence and development of CP. Using ROS immunofluorescence labeling and biochemical kits, we found that the expression of Green Fluorescent Protein-ROS (GFP-ROS) in the prostate tissue of rats in the Model group was significantly higher than that in the Control group (Figure 10). At the same time, the expression of MDA in the model group was significantly increased (*P* < 0.05), while the expression of SOD and CAT was significantly decreased (*P* < 0.05) (Figure 11). After treatment with QSSMS, GFP-ROS expression decreased significantly in a dose-dependent manner, more obviously in the medium-dose and high-dose groups (Figure 10). At the same time, the expression of MDA was significantly decreased (*P* < 0.05), while that of SOD and CAT was significantly increased (*P* < 0.05) (Figure 11).

### 3.10. QSSMS Decreased the Expression of Immune Factors

The expression of SIgA in rat prostate tissue and IgG in serum in the Model group was significantly higher than that in the Control group (*P* < 0.05). After treatment with QSSMS, their expression decreased in a dose-dependent manner (*P* < 0.05), more obviously in the QSSMS-H group (Figures 11(d) and 11(e)).

## 4. Discussion

Our pharmacological analysis showed that the APIs of QSSMS included quercetin, kaempferol, formononetin, isorhamnetin, and calycosin. Quercetin is a natural flavonoid compound with good anti-inflammatory and antioxidant properties. It can improve cell damage caused by oxidative stress and inhibit COX-2 expression [20, 21]. In CP patients treated with quercetin, pain scores decreased, quality of life scores increased, and the expression of PGE2 and prostaglandin F2 in the patients' prostatic massage fluid decreased [22]. Kaempferol has also antioxidative and anti-inflammatory effects and reduces apoptosis by inhibiting the expression of P53, TGF-*β*_1_, and COX-2 [23]. Formononetin promotes apoptosis of human prostate cancer cells by regulating inflammation based on the MAPK signal transduction pathway and NF-*κ*B pathway [24]. Isorhamnetin can repair the ocular surface injury in mice and downregulate the expression of inflammatory cytokines IL-1*β*, IL-8, and TNF-*α* [25]. Calycosin isoflavones can inhibit the NF-*κ*B signaling pathway and play anti-inflammatory, antioxidative, and immunomodulatory roles [26, 27]. The efficacy of QSSMS may be related to the synergic effects of active substances. The biological processes of QSSMS mainly involve negative regulation of the apoptotic process, oxidation-reduction process, inflammatory response, immune response, and so on. We found that IL6, MAPK3, JUN, and TNF were the main targets of QSSMS. Molecular docking showed that the five APIs could bind to these targets, especially the binding between isorhamnetin and PGE2. Quercetin has been used to treat CP due to its evident anti-inflammatory and antioxidative effects [28, 29]. QSSMS may act on CP through multiple APIs, targets, and pathways.

In clinical practice, CP patients often show aggregation of inflammatory factors in prostatic massage fluid. This condition can be simulated through the injection of carrageenan into the ventral lobe of the prostate [30, 31]. The successful establishment of the model can be judged according to inflammatory cell infiltration, tissue edema, necrosis of glandular epithelium, shedding, and so on. In the present experiment, we used carrageenan injection to construct a rat CP model. After 7 d, the pathological changes were similar to those in Wu's model, with significant lymphocyte infiltration, decreased acinar diameter, dilated glandular lumen, and interstitial edema [32]. After 28 d of QSSMS treatment, we found that the glandular lumen structure of the prostate tissue was significantly repaired, inflammatory cell infiltration reduced, interstitial space narrowed, and fibrocyte proliferation and vascular proliferation were inhibited. At the same time, we also found the higher expression of IL-6, IL-1*β*, and TNF-*α* in the model group. This is consistent with the research results of Hu et al. [33]. However, the expression of IL-6, IL-1*β*, and TNF-*α* decreased significantly after QSSMS treatment, confirming that QSSMS can inhibit inflammation. Cox-2 can promote the synthesis of PGE2 and reduce the expression of *β*-endorphin, thus bringing with an analgesic effect [34]. NGFs can maintain the normal function of the nervous system. The increase of NGFs can regulate the neuronal activity, the release of neurotransmitters, and the immune response [35]. Patients with CP often present with periluminal pain and discomfort, which may be related to activation of the NGF-TRKA pathway [36]. In our study, the expression levels of COX-2, PGE2, and NGF in the model group were significantly higher than those in the control group. After QSSMS treatment, these expression levels decreased. Therefore, QSSMS can relieve not only inflammation in the prostate tissue but also pelvic pain in CP patients by regulating neurons and neurotransmitters.

**Figure 12:**
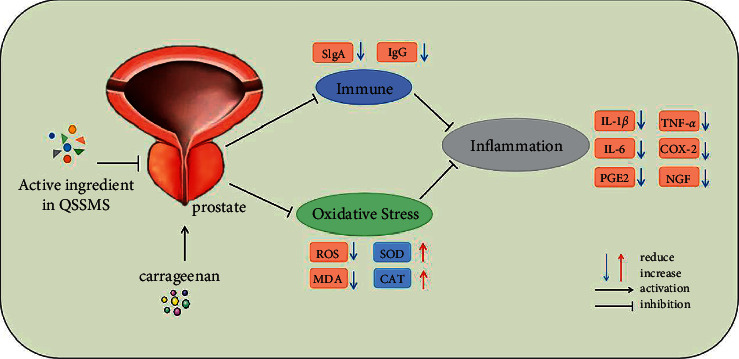
Possible mechanism of QSSMS in treating CP.

Oxidative stress injury has been proven to be closely related to the occurrence and development of CP [37]. MDA is a lipid peroxidation product and one of the markers of oxidative stress damage [38]. SOD and CAT scavenge oxygen-free radicals to protect cells from oxidative stress damage. Basal cell and epithelial cell damage in the prostate can lead to excessive release of inflammatory cells and ROS [39]. Studies have shown that XLQ may treat CP by enhancing the activity of antioxidant enzymes and reducing the water produced by lipid peroxidation [1]. In our study, it was found that the expression levels of ROS and MDA were significantly higher, but those SOD and CAT were significantly lower in the model group than in the control group. After treatment with QSSMS, the expression levels of ROS and MDA decreased significantly, while those of SOD and CAT increased. Therefore, the efficacy of QSSMS may lie in its protection against oxidative stress injury, which is similar to the research results of Meng et al. and Zhao et al. [28, 40]. Moderate immune response can enhance the body's defense function, but the excessive immune response causes damage. Studies have shown that the immune response of serum IgG to SP protein in CP patients is significantly increased, thereby leading to inflammatory cell infiltration in the male reproductive tract and abnormal semen quality [41]. The high expression level of IgG in the serum of CP patients may be an important reason for its inflammatory response [42]. In this study, we found that the expression of SIgA in the prostate tissue and IgG in the serum of rats in the Model group was significantly higher than that in the Control group. After treatment with QSSMS, the expression of SIgA in prostate tissue and IgG in the serum was significantly decreased, which is consistent with the findings of Ye et al. QSSMS functions on CP through repairing oxidative stress injury and regulating the immune system (Figure 12).

## 5. Conclusion

APIs in QSSMS may counteract CP by regulating immunity and reducing oxidative stress injury. QSSMS can improve the microenvironment in the prostate, promote local microcirculation, and inhibit the expression of inflammatory factors. This research provides theoretical support for the application of QSSMS in the clinical treatment of CP.

## Figures and Tables

**Table 1:** Docking results of key APIs and target molecules.

Compound	Binding energy (kcal/mol)				
	IL-1*β*	IL-6	TNF-*α*	COX-2	PGE2
Quercetin	−7.1	−4.5	−8.4	−8.1	−4.7
Kaempferol	−7.2	−6.7	−8	−8.8	−8.7
Formononetin	−7.5	−6.7	−8	−7.8	−8.2
Isorhamnetin	−7.3	−5.1	−8.5	−8.7	−9
Calycosin	0	−6.5	−7.4	−7.9	0

## Data Availability

The data used to support the findings of this study are available from the corresponding author upon request.

## Abbreviations

APIs: Active pharmaceutical ingredients
QSSMS: Qinshi Simiao San
CP: Chronic prostatitis
ER-*β*: Estrogen receptor-*β*
TCMSP: Traditional Chinese Medicine Systems Pharmacology
OB: Oral bioavailability
DL: Drug-likeness
OMIM: Online Mendelian Inheritance in Man
PPI: Protein-protein interaction
STRING: Search Tool for the Retrieval of Interacting Genes/Proteins
GO: Gene Ontology
KEGG: Kyoto Encyclopedia of Genes and Genomes
BP: Biological process
CC: Cellular component
MF: Molecular function
QSSMS-L: Qinshi Simiao San low dose
QSSMS-M: Qinshi Simiao San medium dose
QSSMS-H: Qinshi Simiao San high dose
HE: Hematoxylin and eosin
COX-2: Cyclooxygenase-2
NGF: Nerve growth factor
ROS: Reactive oxygen species
PGE2: Prostaglandin E2
MDA: Malondialdehyde
SOD: Superoxide dismutase
CAT: Catalase
GFP-ROS: Green fluorescent protein-ROS.
